# Identification of Opportunistic Pathogenic Bacteria in Drinking Water Samples of Different Rural Health Centers and Their Clinical Impacts on Humans

**DOI:** 10.1155/2013/348250

**Published:** 2013-06-03

**Authors:** Pavan Kumar Pindi, P. Raghuveer Yadav, A. Shiva Shanker

**Affiliations:** Department of Microbiology, Palamuru University, Mahabubnagar, Andhra Pradesh 509001, India

## Abstract

International drinking water quality monitoring programs have been established in order to prevent or to reduce the risk of contracting water-related infections. A survey was performed on groundwater-derived drinking water from 13 different hospitals in the Mahabubnagar District. A total of 55 bacterial strains were isolated which belonged to both gram-positive and gram-negative bacteria. All the taxa were identified based on the 16S rRNA gene sequence analysis based on which they are phylogenetically close to 27 different taxa. Many of the strains are closely related to their phylogenetic neighbors and exhibit from 98.4 to 100% sequence similarity at the 16S rRNA gene sequence level. The most common group was similar to *Acinetobacter junii* (21.8%) and *Acinetobacter calcoaceticus* (10.9%) which were shared by 7 and 5 water samples, respectively. Out of 55 isolates, only 3 isolates belonged to coliform group which are *Citrobacter freundii* and *Pantoea anthophila*. More than half (52.7%, 29 strains) of the phylogenetic neighbors which belonged to 12 groups were reported to be pathogenic and isolated from clinical specimens. Out of 27 representative taxa are affiliated have eight representative genera in drinking water except for those affiliated with the genera *Exiguobacterium, Delftia, Kocuria,* and *Lysinibacillus*.

## 1. Introduction

Human population growth wields several and various pressures on the quality and the quantity of drinking and fresh water resources and on the access to them. Safe drinking water remains inaccessible to several millions people in the globe. Safe drinking water, thus, reduces the load of waterborne diseases. Contamination of drinking water due to natural and manmade contaminants is frequently reported in developing countries where mainstream of the inhabitants survive in countryside and uptown areas with meager hygiene and waste clearance practices. In developing countries, poor water quality is the most important risk of child mortalities which are mainly through infectious diarrhea. In India, about 10% of the countryside and city populations do not have access to usual safe drinking water and several others are threatened [1].

Potable or drinking water is defined as having a satisfactory quality in terms of its physical, chemical, and bacteriological parameters so that it can be securely used for drinking and cooking. The most common and widespread health risks associated with drinking water in developing countries are of biological origin. The WHO estimates that about 1100 million people globally drink unsafe water, and the greater part of diarrheal disease in the globe (88%) is attributable to insecure water, sanitation, and hygiene [2]. Ten major waterborne diseases are responsible for over 28 billion disease episodes annually in developing countries. The quality of drinking water might be ascertained by its microbiological examination. The maximum threat from microbes (coliform) in water is related to consumption of drinking water, that is, contaminated with human or animal excreta. Coliform bacteria include members of the family *Enterobacteriaceae*, for example, *Escherichia coli*,* Enterobacter aerogenes*,* Salmonella, *and* Klebsiella *in water that are accountable for a variety of diseases like cholera, typhoid, dysenteries, bacillary dysentery, and so forth in human and livestock [3].

Recently, Suthar et al. [4, 5] reported a wide range of pathogenic bacteria in potable water samples from some rural habitations of Northern Rajasthan, India. A total of ten bacterial species, *E. coli*, *Pseudomonas aeruginosa*, *E. aerogenes*, *Klebsiella *sp., *Proteus vulgaris*, *Alcaligenes faecalis*, *Bacillus cereus*, *Staphylococcus aureus*, *Streptococcus lactis*, and *Micrococcus luteus*, were isolated and identified from the potable water samples from this region [4] *M. luteus*, *S. lactis*, *Klebsiella *sp., and *E. coli* were the dominant microflora (recorded from 73.1% of the villages/towns) in the water samples. The presence of coliforms shows the danger of fecal pollution and a consequent hazard of contracting a disease through pathogenic organisms. Nonetheless, the disease-causing organisms (pathogens) mostly transmitted via drinking water are predominantly of fecal origin.

The results of the recent study on the detection of potentially pathogenic bacteria in the drinking water distribution system of a hospital in Hungary by Felföldi et al. [5] had shown the presence of *Legionella*, *Pseudomonas aeruginosa,* and also several other opportunistic pathogenic bacteria, such as *Escherichia albertii*, *Acinetobacter lwoffii* and *Corynebacterium tuberculostearicum*, emphasizing that drinking water systems, particularly those with stagnant water sections, could be the source of nosocomial infections.

Hospitals drinking (potable) water systems are the most important and controllable as well as the most overlooked source of nosocomial pathogens. Conventional culture-based microbiological water quality monitoring techniques take a long time (several days), and usually a small volume of water is sampled (typically 100 mL), which gives rise to inadequate detection limits with regard to drinking water safety. Furthermore, the presence of some important waterborne pathogens (such as *Pseudomonas aeruginosa* or *legionellae*) shows no correlation with conventional indicator organism counts. Water-related pathogens can also find niches in water systems (i.e., an association with biofilms or free-living amoebae), rendering their observation with conventional techniques more difficult. Molecular techniques provide new and rapid facilities for the detection of pathogens involved in nosocomial infections. Five representative end-points (taps) in the drinking water system of a hospital (Budapest, Hungary) were sampled in October 2005.

## 2. Materials and Methods

### 2.1. Source of Samples

Thirteen drinking water samples were collected from different government hospitals, Mahabubnagar District, Andhra Pradesh, India, on 22nd and 23rd of November, 2009. Fifty five strains were isolated from these 13 drinking water sources on nutrient agar medium at 37°C. For isolation of bacteria, 100 *μ*L of the water sample was plated on nutrient agar medium and incubated at 37°C for 2 days. Based on the different colony morphology from each sample, a total of 55 strains were selected and characterized in the present study.

### 2.2. 16S rRNA Gene Sequencing and Phylogenetic Analysis

For 16S rRNA gene sequencing, DNA was prepared using the Mo Bio microbial DNA isolation kit (Mo Bio Laboratories Inc., Solano Beach, CA, USA) and sequenced as described previously [6]. The resultant almost complete sequence of the 16S rRNA gene contained 1502 nucleotides. The 16S rRNA gene sequence of the isolate was subjected to BLAST sequence similarity search [7] and EzTaxon [8] to identify the nearest taxa. The entire related 16S rRNA gene sequences were downloaded from the database (http://www.ncbi.nlm.nih.gov/) aligned using the CLUSTAL_X program [9] and the alignment corrected manually. Phylogenetic tree was constructed using tree-making algorithm and the Maximum likelihood (ML) using the PhyML program [10].

### 2.3. Biochemical Tests

The biochemical tests were performed for all the strains obtained by using the HiMViCTM Biochemical Test Kit (HIMEDIA-KB001) and HiAssorted Biochemical Test Kit (HIMEDIA-KB002).

## 3. Results and Discussion

### 3.1. Characterization of the Bacterial Strains from Drinking Water

All these isolates were from different drinking water sources, and all are mesophilic and could grow in the temperature range of 20 to 40°C with optimum growth temperature of 37°C. All the strains could grow without NaCl in the medium, and the tolerance to NaCl varied from 1 to 2%. All strains could grow either in the pH range of 6–8. 

### 3.2. Identification of Isolated Strains from Drinking Water

**Figure 1:**
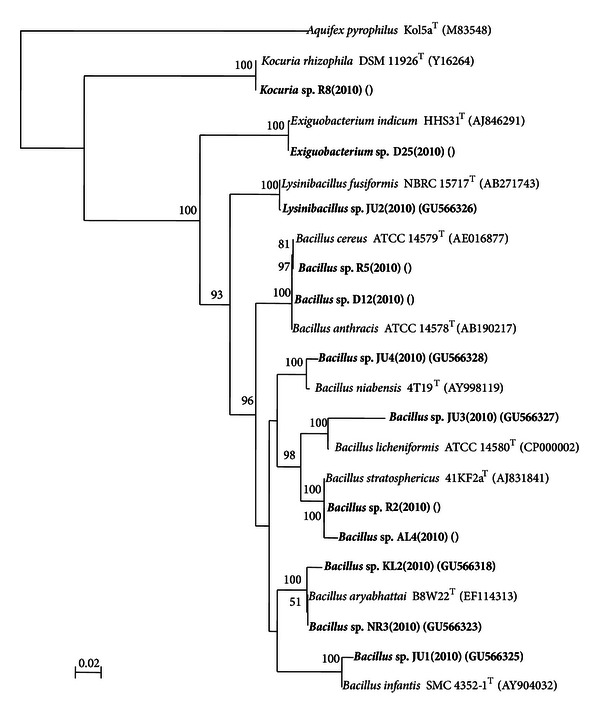
The affiliation of the strains to the nearest phylogenetic neighbor and the percentage of 16S rRNA gene sequence similarities.

**Figure 2:**
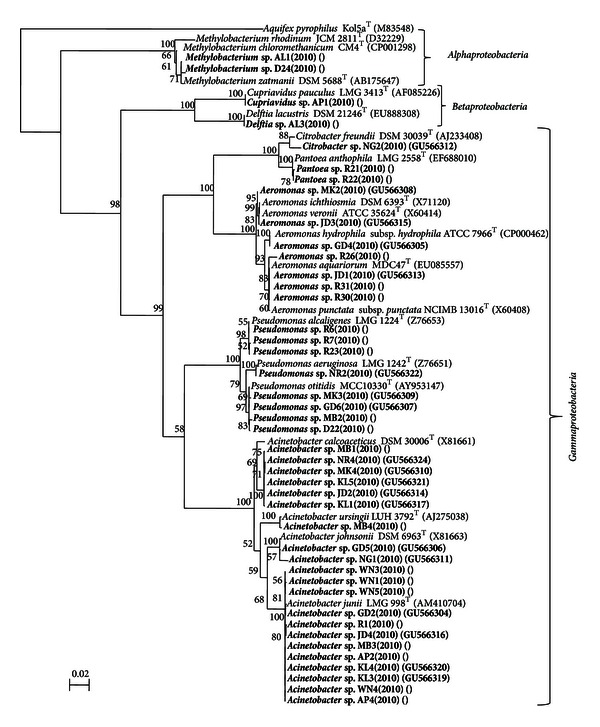
The affiliation of the strains to the nearest phylogenetic neighbor and the percentage of 16S rRNA gene sequence similarities.

A total of 55 bacterial strains were recovered from the 13 drinking water sources (Table 1). Taxonomic analysis of all the 55 strains isolated from water samples indicated that 43 strains were Gram-negative and 12 Gram-positive. The nearest phylogenetic neighbor of all 55 isolated strains was identified following BLAST analysis of the 16S rRNA gene sequence. BLAST analysis indicated that twenty one strains belonged to the genus *Acinetobacter*, nine strains belonged to the genus *Bacillus*, eight strains belonged to the genus *Pseudomonas*, seven strains to the genus *Aeromonas*, two strains each belonged to the genera *Methylobacterium* and *Pantoea,* and one strain belonged to the genera *Cupriavidus*,* Delftia*, *Exiguobacterium*, *Kocuria, *and* Lysinibacillus, *respectively (Table 2). A total 27 different taxa were identified based on the BLAST analysis out of 55 strains. The most common group was similar to *Acinetobacter junii* (21.8%) and *Acinetobacter calcoaceticus* (10.9%) which were shared by 7 and 5 water samples, respectively. The phylogenetic trees constructed to determine their affiliations are shown in Figures 1 and 2. Out of 12 Gram-positive groups, 11 belonged to the phylum *Firmicutes* and 1 belonged to the phylum *Actinobacteria* (Table 2 and Figure 2). All 43 Gram-negative groups were belonged to the phylum *Proteobacteria*. Among the *Proteobacteria*, the *Alphaproteobacteria*, *Betaproteobacteria*, and *Gammaproteobacteria *were represented by 2, 2, and 39 groups, respectively (Table 2 and Figure 2). Phylogenetic analysis based on 16S rRNA gene sequences indicated that the 21 *Acinetobacter* strains belonged to 4 groups, 9 *Bacillus* strains belonged to 7 groups, 8 *Pseudomonas* strains belonged to 3 groups, 7 *Aeromonas* strains belonged to 5 groups, and 2 *Methylobacterium* strains formed two distinct groups (Figures 1 and 2). The affiliation of the strains to the nearest phylogenetic neighbor and the percentage of 16S rRNA gene sequence similarities are indicated in Table 2. Many of the strains are closely related to one another and exhibit 98.4 to 100% sequence similarity at the 16S rRNA gene sequence level like KL-1 and JD-2 which are closely related to one another (100%) and are affiliated to *Acinetobacter calcoaceticus* DSM 30006^T^  (98.5%).

### 3.3. Biochemical Assay

Biochemical analysis of the genus *Bacillus* gave positive results for Indole, Citrate, Oxidase, and Urease and positive results for Methyl red Voges-Proskaeur, Catalase, and Gelatinase tests. Genus *Methylobacterium* gave positive results for Citrate, Catalase, and Urease and negative results for Indole, Methyl Red, Voges-Proskaeur, Oxidase, and Gelatinase. Genus *Cupriavidus* gave positive results for Citrate, Oxidase, Catalase, and Urease and negative results for Indole, Methyl red, Voges-Proskaeur, and Gelatinase. *Delftia* gave positive results for Citrate and Urease and negative ones for Indole, Methyl red, Voges-Proskaeur, Oxidase, Catalase, and Gelatinase. Genus *Acinetobacter* gave positive results for Citrate and Gelatinas and negative results for Indole, Methyl red, Voges-Proskaeur, Oxidase, Catalase, and Urease (*A. ursingii* gave positive results even for Citrate and Oxidase and Catalase). Genus *Aeromonas* (*Ar. aquariorum* gave positive results for all except Catalase and Gelatinase, whereas *Ar. hydrophila*, *Ar. Ichthiosmia*, and *Ar*. *punctata* gave positive results for all tests except Methyl red). Genus *Citrobacter* gave positive results for Methyl red, Oxidase, Catalase, and Urease and negative results for Indole, VP, Citrate, and Gelatinase. Genus *Pantoea* gave positive results for VP, Citrate, and Catalase and negative results for Indole, Methyl red, Oxidase, Urease, and Gelatinase. Genus *Pseudomonas* gave positive results for Citrate, Oxidase, Catalase, and Gelatinase and negative ones for Indole, Methyl red, VP, and Urease. Kocuria rhizophila gave positive results for Oxidase and Catalase and negative ones for the remaining tests. *Exiguobacterium indicum* gave positive results for Methyl red, Citrate, and Catalase and negative ones for Indole,VP, Oxidase, Urease, and Gelatinase. *Lysinibacillus fusiformis* gave positive results for Oxidase and Gelatinase and negative results for the remaining tests (Table 3).

Coliform is the name of a test adopted in 1914 by the Public Health Service for the *Enterobacteriaceae* family. It is the commonly used bacterial indicator of sanitary quality of foods and water. Coliform bacteria live in soil or vegetation and in the gastrointestinal tract of animals. Coliform bacteria enter water supplies from the direct disposal of waste into streams or lakes, or from runoff, from wooded areas, pastures, feedlots, septic tanks, and sewage plants into streams or groundwater. Coliform bacteria are not a single type of bacteria, but a group of bacteria that includes many strains, such as *E*. *coli*. They are ubiquitous in nature, and many types are harmless. Coliform bacteria belong to the genera *Citrobacter*, *Enterobacter*, *Escherichia*, *Hafnia*, *Klebsiella*, *Pantoea*, and *Serratia*. Out of 55 isolates only 3 isolates belonged to coliform group which are NG2 which is phylogenetically close to *Citrobacter freundii* and R21 and R22 which are phylogenetically close to *Pantoea anthophila*. This shows that the water samples NG and R collected from Nagarkurnool and Mahabubnagar-2 might be contaminated with fecal pollution and a consequent hazard of contracting a disease through pathogenic organisms. Nonetheless, the disease-causing organisms (pathogens) mostly transmitted via drinking water are predominantly of fecal origin. A total of 2 and 12 strains were isolated from the samples NG and R which include 3 coliform bacteria, and out of 14 strains, 7 were likely to be pathogenic which include NG1 which is phylogenetically close to *Acinetobacter johnsonii*, R1 which is phylogenetically close to *Acinetobacter junii*, NG2 which is phylogenetically close to *Citrobacter freundii*, R6, 7, and 23 which are phylogenetically close to *Pseudomonas alcaligenes*, and R5 which is phylogenetically close to* Bacillus cereus*.

In the present study, species that belonged to the genera *Aeromonas*, *Methylobacterium*, *Bacillus*, and *Pseudomonas* were isolated which is supported by the different studies where several novel species which belong to the genera *Aeromonas* [11], *Methylobacterium* [12], *Bacillus* [13], and* Pseudomonas *[14] were reported from drinking water systems. *Aeromonas *spp. are ubiquitous bacteria found in diverse aquatic environments worldwide such as bottled water, chlorinated water, well water, and heavily polluted waters. *Aeromonas hydrophila* survives easily in waters polluted by feces and seems resistant to various disinfectants, insecticides, and chemicals.

Recent studies on drinking water in rural areas in Northern Rajasthan, India, [4] and in drinking water distribution system of a hospital in Hungary, [5] showed the presence of several bacteria in the drinking water which included *Acinetobacter lwoffii*, *Alcaligenes faecalis*, *Bacillus cereus*, *Corynebacterium tuberculostearicum*, *Enterobacter aerogenes*, *Escherichia coli*, *Escherichia albertii*, *Klebsiella *sp., *Micrococcus luteus*, *Proteus vulgaris*, *Pseudomonas aeruginosa*, *Staphylococcus aureus*, and *Streptococcus lactis*. In the present study, species belonging to the genus *Acinetobacter*, *Bacillus cereus*, and *Pseudomonas aeruginosa* which are opportunistic pathogens were isolated from 9 drinking water samples such as AP, GD, JD, KL, MB, NG, NR, R, and WN out of 13.

Of the 27 groups, only few (GD4, R5, AP1, and NR2) of the phylogenetic neighbors had been isolated earlier from the drinking water sources including *Aeromonas hydrophila* [4], *Bacillus cereus* [4], *Cupriavidus pauculus* [15], and* Pseudomonas aeruginosa* [4, 5]. More than half (52.7%, 29 strains) of the phylogenetic neighbors which belonged to 12 groups were reported to be pathogenic and were isolated from clinical specimens, and other 15 groups were reported to be isolated from several distinct habitats. Most of the drinking water sources studied for bacterial diversity include bacteria which are phylogenetically close to pathogenic bacteria except one sample AL from which 3 strains AL1, AL3, and AL4 were isolated which belong to the phylogenetic neighbors *Methylobacterium zatmanii*, *Delftia lacustris*, and *Bacillus stratosphericus*, respectively. The isolates which are likely to be pathogenic include strain AP1 which is phylogenetically close to *Cupriavidus pauculus* which causes human infections sporadically and were isolated earlier from a variety of human clinical sources and also from drinking water sources [15] (Table 4); strains GD5 and NG1 which are phylogenetically close to *Acinetobacter johnsonii* which causes vascular catheter-related bloodstream infection were isolated from clinical specimens and milk [16] (Table 4); strains AP2, AP4, GD2, JD4, KL3, KL4, MB3, R1, WN1, WN3, WN4, and WN5 which are phylogenetically close to *Acinetobacter junii* which causes septicemia, meningitis, peritonitis, and so forth were isolated from human clinical specimens like urine [17] (Table 4); strain MB4 which is phylogenetically close to *Acinetobacter ursingii* which causes bacteremia was isolated from blood [18] (Table 4); strain GD4 which is phylogenetically close to *Aeromonas hydrophila* subsp. *Hydrophila* which causes acute diarrheal disease was isolated from humans, animals, fish, and fresh water [11, 19] (Table 4); strain NG2 which is phylogenetically close to *Citrobacter freundii* which causes opportunistic infections was isolated from canal water [20] (Table 4); strain NR2 which is phylogenetically close to *Pseudomonas aeruginosa *which causes several infections in immunocompromised individuals was isolated from wide variety habitats including clinical samples and also from drinking water samples [4, 5] (Table 4); strains R6, R7, and R23 which are phylogenetically close to *Pseudomonas alcaligenes* which causes endocarditis occasionally were isolated from soil, water, and also from clinical samples [21] (Table 4); strains D22, GD6, MB2, and MK3 which are phylogenetically close to *Pseudomonas otitidis* which causes inflammation of the ear were isolated from the ear [22] (Table 4); strain D12 which is phylogenetically close to *Bacillus anthracis* which causes anthrax disease was isolated from sheep blood (Table 4); strain R5 which is phylogenetically close to *Bacillus cereus* which causes food poisoning was isolated from the soil and also food materials [23] (Table 4); and strain JU1 which is phylogenetically close to *Bacillus infantis* which causes sepsis was isolated from clinical specimens [24] (Table 4).

## 4. Conclusion

 This study is an overview of drinking water quality in the rural and urban parts of Mahabubnagar District, India. Recent investigations on bacterial contaminations of drinking water in the hospitals suggested risks of secondary infection to the patients, staff, and also patient relatives. These observations were just a drinking water quality assessment, and no data on human health scenario in this region were recorded. Further detailed studies on the health issues of the patients and other people using this contaminated drinking water are still required to trace the impact of this water consumption on people in those hospitals. The lack of awareness about good sanitation and personal hygienic practices, among governmental rural and also urban hospitals is an important factor for poor drinking water quality in these sources. 

## Figures and Tables

**Table 1:** Bacterial abundance from the drinking water samples collected from government hospitals, Mahabubnagar.

Serial number	Sample number	Place of sample collection	Number of isolates	Isolated strains
1	AL	Alampur	3	AL1, AL3, AL4
2	AP	Achampet	3	AP1, AP2, AP4
3	D	Shadnagar	4	D12, D22, D24, D25
4	GD	Gadwal	4	GD2, GD4–D6
5	JD	Jadcherla	4	JD1–JD4
6	JU	Jurala	4	JU1–JU4
7	KL	Kalwakurthy	5	KL1–KL5
8	MB	Mahabubnagar-1	4	MB1–MB4
9	MK	Makthal	3	MK2–MK4
10	NG	Nagar Kurnool	2	NG1, NG2
11	NR	Narayanpet	3	NR2–NR4
12	R	Mahabubnagar-2	12	R1, R2, R5–R8, R21–R23, R26, R30, R31
13	WN	Wanaparthy	4	WN1, WN3–WN5

**Table 2:** Identification of the 55 bacterial strains isolated from the drinking water samples collected from different government hospitals, Mahabubnagar, based on BLAST analysis of the 16S rRNA gene sequences.

Sl no.	Strain no.	Nearest phylogenetic neighbor	16S rRNA gene sequence similarity %
		Gram-negative bacterial strains	

		*Proteobacteria *	
		*Alphaproteobacteria *	
1	D24	*Methylobacterium chloromethanicum *CM4^T^ CP001298	99.6
2	AL1	*Methylobacterium zatmanii *DSM 5688^T^ AB175647	99.7
		*Betaproteobacteria *	
3	AP1	*Cupriavidus pauculus *LMG 3413^T^ AF085226	100.0
4	AL3	*Delftia lacustris *DSM 21246^T^ EU888308	100.0
		*Gammaproteobacteria *	
5	JD2	*Acinetobacter calcoaceticus *DSM 30006^T^ X81661	98.5
6	KL1	*Acinetobacter calcoaceticus *DSM 30006^T^ X81661	98.5
7	KL5	*Acinetobacter calcoaceticus *DSM 30006^T^ X81661	98.5
8	MB1	*Acinetobacter calcoaceticus *DSM 30006^T^ X81661	98.4
9	MK4	*Acinetobacter calcoaceticus *DSM 30006^T^ X81661	98.5
10	NR4	*Acinetobacter calcoaceticus *DSM 30006^T^ X81661	98.4
11	GD5	*Acinetobacter johnsonii *DSM 6963^T^ X81663	99.9
12	NG1	*Acinetobacter johnsonii *DSM 6963^T^ X81663	99.5
13	AP2	*Acinetobacter junii *LMG 998^T^ AM410704	100.0
14	AP4	*Acinetobacter junii *LMG 998^T^ AM410704	100.0
15	GD2	*Acinetobacter junii *LMG 998^T^ AM410704	100.0
16	JD4	*Acinetobacter junii *LMG 998^T^ AM410704	100.0
17	KL3	*Acinetobacter junii *LMG 998^T^ AM410704	100.0
18	KL4	*Acinetobacter junii *LMG 998^T^ AM410704	100.0
19	MB3	*Acinetobacter junii *LMG 998^T^ AM410704	100.0
20	R1	*Acinetobacter junii *LMG 998^T^ AM410704	99.9
21	WN1	*Acinetobacter junii *LMG 998^T^ AM410704	99.9
22	WN3	*Acinetobacter junii *LMG 998^T^ AM410704	100.0
23	WN4	*Acinetobacter junii *LMG 998^T^ AM410704	100.0
24	WN5	*Acinetobacter junii *LMG 998^T^ AM410704	99.9
25	MB4	*Acinetobacter ursingii *LUH 3792^T^ AJ275038	99.6
26	JD1	*Aeromonas aquariorum *MDC47^T^ EU085557	100.0
27	R31	*Aeromonas aquariorum *MDC47^T^ EU085557	100.0
28	R30	*Aeromonas aquariorum *MDC47^T^ EU085557	99.9
29	GD4	*Aeromonas hydrophila *subsp.* hydrophila *ATCC 7966^T^ CP000462	100.0
30	JD3	*Aeromonas ichthiosmia *DSM 6393^T^ X71120	99.8
31	MK2	*Aeromonas ichthiosmia *DSM 6393^T^ X71120	99.8
32	R26	*Aeromonas punctata *subsp.* punctata *NCIMB 13016^T^ X60408	99.2
33	NG2	*Citrobacter freundii *DSM 30039^T^ AJ233408	98.3
34	R21	*Pantoea anthophila *LMG 2558^T^ EF688010	99.8
35	R22	*Pantoea anthophila *LMG 2558^T^ EF688010	99.9
36	NR2	*Pseudomonas aeruginosa *LMG 1242^T^ Z76651	99.9
37	R6	*Pseudomonas alcaligenes *LMG 1224^T^ Z76653	100.0
38	R7	*Pseudomonas alcaligenes *LMG 1224^T^ Z76653	99.8
39	R23	*Pseudomonas alcaligenes *LMG 1224^T^ Z76653	99.8
40	D22	*Pseudomonas otitidis *MCC10330^T^ AY953147	99.8
41	GD6	*Pseudomonas otitidis *MCC10330^T^ AY953147	99.8
42	MB2	*Pseudomonas otitidis *MCC10330^T^ AY953147	99.6
43	MK3	*Pseudomonas otitidis *MCC10330^T^ AY953147	99.7

		Gram-positive bacterial strains	

		*Actinobacteria *	
44	R8	*Kocuria rhizophila *DSM 11926^T^ Y16264	100.0
		*Firmicutes *	
		*Bacilli *	
45	D12	*Bacillus anthracis *ATCC 14578^T^ AB190217	100.0
46	KL2	*Bacillus aryabhattai *B8W22^T^ EF114313	98.7
47	NR3	*Bacillus aryabhattai *B8W22^T^ EF114313	99.7
48	R5	*Bacillus cereus *ATCC 14579^T^ AE016877	100.0
49	JU1	*Bacillus infantis *SMC 4352-1^T^ AY904032	99.5
50	JU3	*Bacillus licheniformis *ATCC 14580^T^ CP000002	99.9
51	JU4	*Bacillus niabensis *4T19^T^ AY998119	98.9
52	AL4	*Bacillus stratosphericus *41KF2a^T^ AJ831841	99.9
53	R2	*Bacillus stratosphericus *41KF2a^T^ AJ831841	100.0
54	D25	*Exiguobacterium indicum HHS31* ^ T^ AJ846291	99.9
55	JU2	*Lysinibacillus fusiformis *NBRC 15717^T^ AB271743	99.8

The accession numbers of the 55 strains are GU566304 and GU566358.

**Table 3:** Biochemical tests for the strains obtained.

Sl no.	Strain no.	Nearest phylogenetic neighbor	Biochemical tests							
			Ind	MR	VP	Cit	Oxi	Cat	Ure	Gel
1	D24	*Methylobacterium chloromethanicum* CM4^T^ CP001298	−	−	−	+	−	+	+	−
2	AL1	*Methylobacterium zatmanii* DSM 5688^T ^AB175647	−	−	−	+	−	−	+	−
3	AP1	*Cupriavidus pauculus* LMG 3413^T ^AF085226	−	−	−	+	+	+	+	−
4	AL3	*Delftia lacustris *DSM 21246^T ^EU888308	−	−	−	+	−	−	+	−
5	JD2	*Acinetobacter calcoaceticus *DSM 30006^T^ X81661	−	−	−	+	−	−	+	+
6	KL1	*Acinetobacter calcoaceticus *DSM 30006^T^ X81661	−	−	−	+	−	−	−	+
7	KL5	*Acinetobacter calcoaceticus *DSM 30006^T^ X81661	−	−	−	+	−	−	−	+
8	MB1	*Acinetobacter calcoaceticus *DSM 30006^T^ X81661	−	−	−	+	−	−	−	+
9	MK4	*Acinetobacter calcoaceticus *DSM 30006^T^ X81661	−	−	−	+	−	−	−	+
10	NR4	*Acinetobacter calcoaceticus *DSM 30006^T^ X81661	−	−	−	+	−	−	−	+
11	GD5	*Acinetobacter johnsonii *DSM 6963^T^ X81663	−	−	−	−	−	−	−	−
12	NG1	*Acinetobacter johnsonii *DSM 6963^T^ X81663	−	−	−	−	−	−	−	−
13	AP2	*Acinetobacter junii * LMG998^T^ AM410704	−	−	−	−	−	−	−	−
14	AP4	*Acinetobacter junii* LMG998^T^ AM410704	−	−	−	−	−	−	−	−
15	GD2	*Acinetobacter junii* LMG998^T^ AM410704	−	−	−	−	−	−	−	−
16	JD4	*Acinetobacter junii* LMG998^T^ AM410704	−	−	−	−	−	−	−	−
17	KL3	*Acinetobacter junii* LMG998^T^ AM410704	−	−	−	−	−	−	−	−
18	KL4	*Acinetobacter junii* LMG998^T^ AM410704	−	−	−	−	−	−	−	−
19	MB3	*Acinetobacter junii* LMG998^T^ AM410704	−	−	−	−	−	−	−	−
20	R1	*Acinetobacter junii* LMG998^T^ AM410704	−	−	−	−	−	−	−	−
21	WN1	*Acinetobacter junii* LMG998^T^ AM410704	−	−	−	−	−	−	−	−
22	WN3	*Acinetobacter junii* LMG998^T^ AM410704	−	−	−	−	−	−	−	−
23	WN4	*Acinetobacter junii* LMG998^T^ AM410704	−	−	−	−	−	−	−	−
24	WN5	*Acinetobacter junii* LMG998^T^ AM410704	−	−	−	−	−	−	−	−
25	MB4	*Acinetobacter ursingii *LUH 3792^T^ AJ275038	−	−	−	+	+	+	−	−
26	JD1	*Aeromonas aquariorum* MDC47^T^ EU085557	+	+	+	+	+	−	+	−
27	R31	*Aeromonas aquariorum* MDC47^T^ EU085557	+	+	+	+	+	−	+	−
28	R30	*Aeromonas aquariorum* MDC47^T ^EU085557	+	+	+	+	+	−	+	−
29	GD4	*Aeromonas hydrophila *subsp.* hydrophila* ATCC 7966^T^ CP000462	+	−	+	+	+	+	+	+
30	JD3	*Aeromonas ichthiosmia *DSM 6393^T^ X71120	+	−	+	+	+	+	+	+
31	MK2	*Aeromonas ichthiosmia *DSM 6393^T ^X71120	+	−	+	+	+	+	+	+
32	R26	*Aeromonas punctata* subsp. *punctate *NCIMB 13016^T^ X60408	+	−	+	+	+	+	+	+
33	NG2	*Citrobacter freundii *DSM 30039^T ^AJ233408	−	+	−	−	+	+	+	−
34	R21	*Pantoea anthophila* LMG2558^T ^EF688010	−	−	+	+	−	+	−	−
35	R22	*Pantoea anthophila* LMG2558^T^ EF688010	−	−	+	+	−	+	−	−
36	NR2	*Pseudomonas aeruginosa *LMG 1242^T^ Z76651	−	−	−	+	+	+	−	+
37	R6	*Pseudomonas alcaligenes *LMG 1224^T^ Z76653	−	−	−	+	+	+	−	+
38	R7	*Pseudomonas alcaligenes *LMG 1224^T^ Z76653	−	−	−	+	+	+	−	+
39	R23	*Pseudomonas alcaligenes *LMG 1224^T^ Z76653	−	−	−	+	+	+	−	+
40	D22	*Pseudomonas otitidis* MCC10330^T ^AY953147	−	−	−	+	+	+	−	+
41	GD6	*Pseudomonas otitidis* MCC10330^T^ AY953147	−	−	−	+	+	+	−	+
42	MB2	*Pseudomonas otitidis* MCC10330^T^ AY953147	−	−	−	+	+	+	−	+
43	MK3	*Pseudomonas otitidis* MCC10330^T^ AY953147	−	−	−	+	+	+	−	+
44	R8	*Kocuria rhizophila *DSM 11926^T^ Y16264	−	−	−	−	+	+	−	−
45	D12	*Bacillus anthracis *ATCC 14578^T^ AB190217	−	+	+	−	−	+	−	+
46	KL2	*Bacillus aryabhattai* B8W22^T^ EF114313	−	+	+	−	−	+	−	+
47	NR3	*Bacillus aryabhattai* B8W22^T^ EF114313	−	+	+	−	−	+	−	+
48	R5	*Bacillus cereus *ATCC 14579^T^ AE016877	−	+	+	−	−	+	−	+
49	JU1	*Bacillus infantis* SMC4352-1^T ^AY904032	−	+	+	−	−	+	−	+
50	JU3	*Bacillus licheniformis* ATCC 14580 CP000002	−	+	+	−	−	+	−	+
51	JU4	*Bacillus niabensis* 4T19^T^ AY998119	−	+	+	−	−	+	−	+
52	AL4	Bacillus stratosphericus 41KF2a^T ^AJ831841	−	+	+	−	−	+	−	+
53	R2	*Bacillus stratosphericus* 41KF2a^T ^AJ831841	−	+	+	−	−	+	−	+
54	D25	*Exiguobacterium indicum HHS31* ^ T ^AJ846291	−	+	−	+	−	+	−	−
55	JU2	*Lysinibacillus fusiformis* NBRC 15717^T ^AB271743	−	−	−	−	+	−	−	+

Ind: Indole; MR: Methyl red; VP: Voges Proskeur; Cit: Citrate; Oxi: Oxidase; Cat: Catalase; Ure: Urease; and Gel: Gelatinase.

**Table 4:** Isolation of source of the type strains associate with disease.

Sl no.	Nearest phylogenetic neighbor	Isolation source	Associated human disease	Reference
1	*Cupriavidus pauculus* LMG 3413^T^ AF085226	Isolated from a variety of human clinical sources including blood, wounds, sputum, urine, eye, throat, and peritoneal fluid. In addition strains have been isolated from pool water, ground water, and bottled mineral water	Sporadically cause human infections	Vandamme et al., 1999 [15]

2	*Acinetobacter johnsonii* DSM 6963^T^ X81663	Isolated from clinical specimens and from eviscerated chickens and may cause ropiness in milk. Isolated from duodenum	Vascular catheter-related bloodstream infection	Seifert et al., 1993 [16]

3	*Acinetobacter junii *LMG 998^T^ AM410704	Isolated from human clinical specimens like urine	Septicemia, community-acquired bacterial meningitis, peritoneal dialysis-related peritonitis, and infections associated with corneal perforation	Hung et al., 2009 [17]

4	*Acinetobacter ursingii* LUH 3792^T^ AJ275038	Isolated from blood of a hospitalized patient with endocarditis	Bacteremia	Loubinoux et al., 2003 [18]

5	*Aeromonas hydrophila *subsp. *hydrophila* ATCC 7966^T^ CP000462	Isolated from humans, animals, fish, and fresh water	Acute diarrheal disease	Ljungh et al., 1977 [19]

6	*Citrobacter freundii *DSM 30039^T^ AJ233408	Isolated from canal water	Opportunistic infections like neonatal meningitis	Badger et al., 1999 [20]

7	*Pseudomonas aeruginosa* LMG 1242^T^ Z76651	Found in soil, water, skin flora, and most manmade environments throughout the world	Localized infection of eye, ear, skin, urinary, and respiratory. Bone, joint, gastrointestinal tract, central nervous system, and systemic infection with bacteremia. Secondary pneumonia. Endocarditis	

8	*Pseudomonas alcaligenes *LMG 1224^T^ Z76653	Common soil and water inhabitant that has rarely been proven a human pathogen	Endocarditis	Valenstein et al., 1983 [21]

9	*Pseudomonas otitidis* MCC 10330^T^ AY953147	Isolated from the ears of patients with acute otitis externa (inflammation of the ear)	Inflammation of the ear	Clark et al., 2006 [22]

10	*Bacillus anthracis *ATCC 14578^T^ AB190217	Isolated from the blood of sheep suffering from anthrax	Cutaneous anthrax, pulmonary anthrax, and gastrointestinal anthrax	

11	*Bacillus cereus *ATCC 14579^T^ AE016877	Isolated from soil and food materials	Food poisoning	Todar, 2008 [23]

12	*Bacillus infantis *SMC 4352-1^T^ AY904032	Isolated from a newborn child with sepsis	Sepsis	Ko et al., 2006 [24]

Out of 27 representative taxa are affiliated have eight representative genera in drinking water except for those affiliated with the genera *Exiguobacterium*, *Delftia*, *Kocuria*, and *Lysinibacillus*.
